# Iron and Manganese Alginate for Rechargeable Battery Electrodes

**DOI:** 10.3390/polym15030639

**Published:** 2023-01-26

**Authors:** Lindah K. Kiriinya, Markus C. Kwakernaak, Simone C. D. Van den Akker, Guy L. M. M. Verbist, Stephen J. Picken, Erik M. Kelder

**Affiliations:** 1 Department of Radiation Science & Technology, Delft University of Technology, 2629 JB Delft, The Netherlands; 2 Department of Electrical & Information Engineering, University of Nairobi, Nairobi 00100, Kenya; 3 Department of Chemical Engineering, Delft University of Technology, 2629 HZ Delft, The Netherlands; 4 Shell Global Solutions Int. BV, 1031 HW Amsterdam, The Netherlands

**Keywords:** electrode material, alginates, rechargeable battery, polysaccharide

## Abstract

We present a sustainable, inherently safe battery chemistry that is based on widely available and cheap materials, that is, iron and manganese hosted in alginate bio-material known from the food and medical industry. The resulting battery can be recycled to allow circularity. The electrodes were synthesised by the alginate caging the multi-valent metals to form a hydrogel in an aqueous environment. Characterisation includes FTIR, XPS and Mössbauer spectroscopy. The electrochemical performance of the electrodes was investigated by performing cyclic voltammetry (CV) and (dis)charge experiments. Mn and Fe ions show good co-ordination with the alginic acid with higher oxidation states demonstrating complex bonding behaviour. The non-optimised iron and manganese alginate electrodes already exhibit a cycling efficiency of 98% and 69%, respectively. This work shows that Fe and Mn atomically disperse in a bio-based host material and can act as electrodes in an aqueous battery chemistry. While demonstrated at cell level, it is furthermore explained how these materials can form the basis for a (semi-solid) flow cell.

## 1. Introduction

The global energy demand is expected to increase by 48% in the next 20 years partly because of the strong growth in world population [1]. Fossil fuels still provide the highest contribution to the world’s energy demand, accounting for 84% of the current energy consumption [2]. There has been a global drive to switch to energy sources that have a lower carbon footprint. Renewable energy presents an opportunity to decarbonise our energy consumption with benefits ranging from a reduction in greenhouse gas emissions to the diversification of energy supplies and a reduced dependency on fossil fuels. However, the fluctuations in electricity demand and supply requires the use of batteries with novel battery chemistries that can provide safe, sustainable and affordable energy storage to overcome this intermittency.

To cope with the above requirements, materials for the negative and positive electrodes need to be low cost, environmental friendly, highly stable and have an acceptable energy density. Organic and bio-based electrodes have gained popularity because of their green and sustainable production and use, as well as their potential for application in large-scale energy storage. This is particularly important once aqueous electrolytes are considered.

More recently, the most studied organic electrode materials have been organic carbonyl compounds. Examples are quinone, naphthalene dianhydride and perylene dianhydride derivatives. When polymerised, these compounds show excellent stability in a lithium-ion battery, providing good capacity [3].

Alginic acid is a polysaccharide that is abundantly available, environmentally friendly, cost effective and nontoxic. It is well-known for its ability to bind multivalent cations, very efficiently forming alginate hydrogels in aqueous environments [4]. This gelation ability has given alginic acid applications in the (bio)medical and food industries [5]. The ability to capture/cage cations inside its structure is of particular interest for battery applications. Iron and manganese ions are attractive candidates for novel electrode materials because of their abundance, low cost and low toxicity, and are well-known for their electron storage ability that is due to the possible changes in oxidation state. Hence, this work investigates the electrochemical properties of iron alginate (Fe-Alg) and manganese alginate (Mn-Alg) for use in rechargeable batteries.

**Figure 1:**
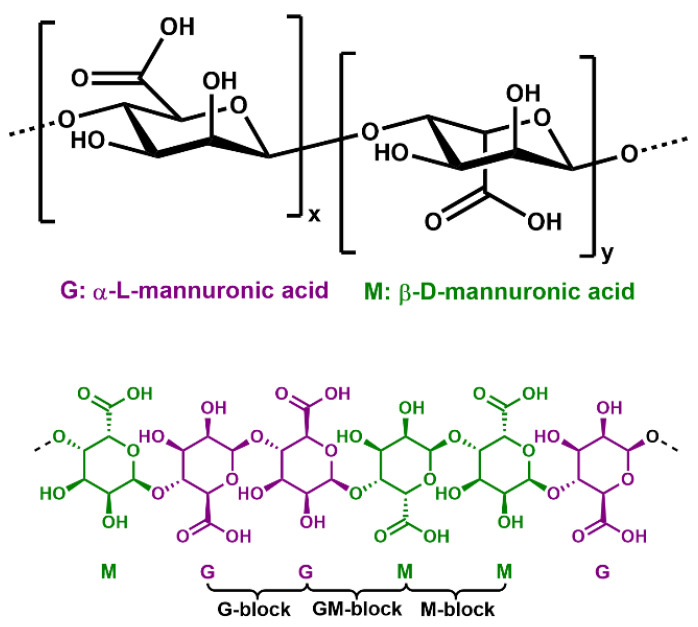
Structural properties of alginate polymers with monomer units: mannuronic (M) and guluronic (G) residues and the configuration of M-, G- and GM-blocks.

Alginic acid is found in the cell walls of brown algae and is composed of two anionic monomers: (1,4) linked α-L guluronic acid (G) and (1,4) linked β-D-mannuronic acid (M) (Figure 1). The carboxyl group in the G- monomer has the same orientation as the hydroxyl group, whereas in the M- monomer the carboxyl group is oriented perpendicular to the hydroxyl group. Upon deprotonation, the negatively charged carboxylate can chelate with cations. Multivalent cations can crosslink the alginate polymer chains. This will increase the viscosity of the solution and in most cases results in the formation of a hydrogel. The cation’s affinity towards the alginate varies depending on its charge, affinity to water, ionic radius and chemical affinity with the alginate. Bivalent alkaline earth cations (Mg^2+^, Ca^2+^ and Sr^2+^) form ionic bonds, whereas bivalent transition metal ions (Mn^2+^, Co^2+^, Cu^2+^, Fe^2+^ and Zn^2+^) and trivalent metal cations (Fe^3+^, Cr^3+^, Al^3+^, Ga^3+^, Sc^3+^ and La^3+^) form complex uronates via strong coordination–covalent bonds [6]. Alginates display a good affinity for transitional metal ions with the affinity being greater for the trivalent metal cations than for divalent cations [6]. Divalent cations tend to bind to two alginate chains. Trivalent cation bonding appears to be more complex; one might expect trivalent cations to bind to three alginate chains. However, some studies have shown that trivalent cations tend to bind to two alginate chains [7,8]. The remaining positive charge is then compensated for by other groups or ions.

**Figure 2:**
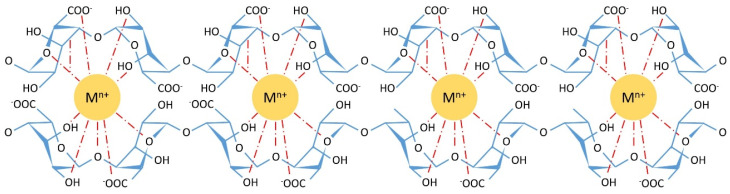
Orientation of a multivalent cation in the egg-box structure; the possible chelate bonds are the dotted red lines.

Differences in the M/G ratio also affect the chelating capability and gel strength [9,10]. Gel formation has been shown to be greatly influenced by the interactions of the G-blocks. The linkage of two G-monomers creates a cavity, making it an ideal place (cage) for a multivalent cation to reside. The crosslinking of the G-blocks by multivalent cations creates a tightly held junction, popularly referred to as ‘egg-box model’ (Figure 2) [11]. Haug et al. experimentally showed that precipitated parts of the alginates are richer in guluronic acid compared to mannuronic acid [11]. Polymers with high M- or MG-block content produce a more elastic and flexible gel while a high G-block content gives stronger and more brittle gels [12]. Cations can also demonstrate preferential bonding to the G and M diads: Sr^2+^ ions only bind to G-blocks, whereas Ca^2+^ binds to G- and GM-blocks and Ba^2+^ only to G- and M-blocks [13]. Here, we will focus on iron(III) alginate (Fe(III)-Alg) and manganese(II) alginate (Mn(II)-Alg).

## 2. Methods and Discussion

### 2.1. Synthesis and Characterisation

#### 2.1.1. Synthesis of Fe(III)-alginate and Mn(II)-alginate

The synthesis of powders of Fe(III)-Alg and Mn(II)-Alg was successfully performed at larger scale. To that end, we used the exchange of the loosely bound sodium ions by multivalent cations. It was noticed that during the synthesis and drying process, it is important to avoid temperatures above 40 °C to minimise alginate decomposition.

In more detail, a solution of sodium alginate (2% *w*/*w*) was added dropwise to a vigorously stirring solution of iron(III)chloride (1 M) or manganese(II)nitrate (1 M). The solution turned into a suspension of fine gel particles and was left to stir for an hour at room temperature. After this, vacuum filtration was used to obtain these gel particles. This was followed by extensive washing of the gel residue with distilled water until the filtrate was colourless. The gel residue was initially dried by washing with ethanol and further dried in a vacuum oven at 30 °C. The dried Fe and Mn alginates were crushed into a powder via ball milling at 240 rotations/min for 5 h (Pulverisette 7, Planetary Micro Mill, Ede, the Netherlands). This yielded the final alginate powders. Reagents for synthesis were purchased at Sigma−Aldrich, Schnelldorf, Germany. Only Manganese(II)nitrate was purchased at Alfa Aesar, Worcester County, MA, USA.

#### 2.1.2. Fourier Transform InfraRed Spectroscopy (FTIR)

**Figure 3:**
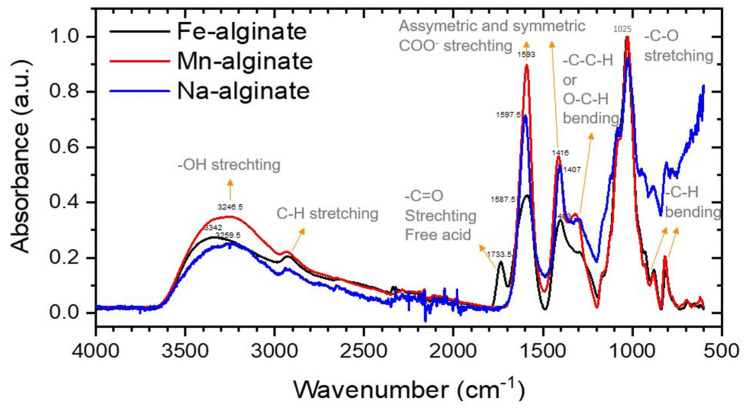
IR spectrum for Fe-Alg-P, Mn-Alg-P and Na-Alg powder. Resolution 2 cm^–1^, 16 scans averaging.

Using FTIR (Frontier FT-IR, PerkinElmer, Waltham, MA, USA) we investigated whether sodium counter-ions were successfully replaced by iron or manganese ions. Spectra were taken for Fe(III)-Alg, Mn(II)-Alg and Na-alginate and these are presented in Figure 3. Several peaks were shifted or changed in intensity compared to Na-alginate, in particular the –OH (~3200 cm^−1^) and -COO^−^(~1400–1600 cm^−1^) bands. In addition, the Fe(III)-Alg shows a new peak that corresponds to the free acid –C=O stretch (~1700 cm^−1^). All these stretch vibrations correspond to bonds that are involved in the binding of the metal to alginate polysaccharide. Although it is not possible to quantify the amount of sodium replacement, it can be observed that it has happened to a major extent. It is further interesting to see that the free –C=O stretch observed for Fe(III)-Alg might indicate a more loosely bound Fe^3+^ ion compared to the other measured divalent ions.

#### 2.1.3. X-ray Photoelectron Spectroscopy (XPS)

**Figure 4:**
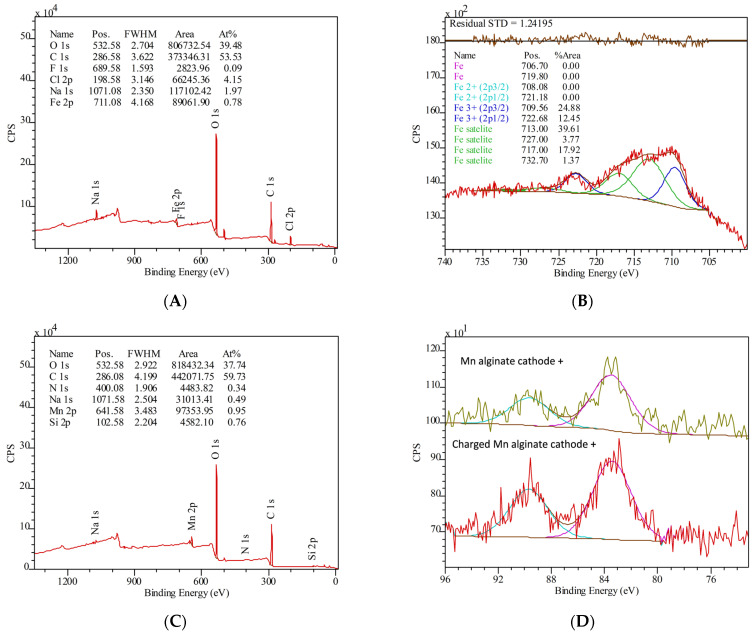
XPS spectra of Fe(III)-Alg (**A**,**B**) and Mn(II)-Alg (**C**,**D**).

**Figure 5:**
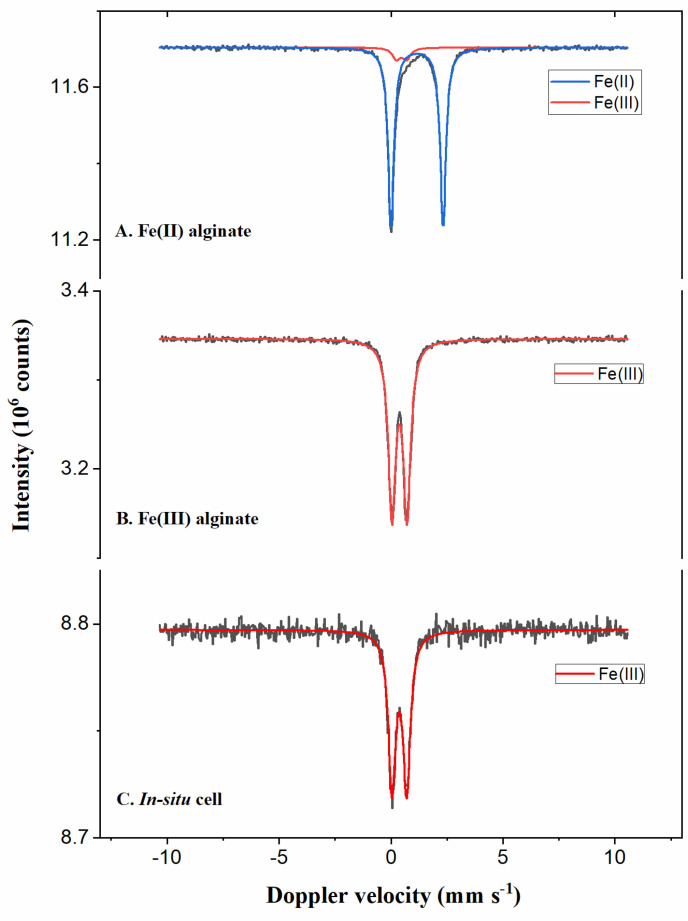
Mössbauer spectra for Fe-Alg powder; (**A**) Fe(II)-Alg powder; (**B**) Fe(III)-Alg powder; (**C**) Fe(III)-Alg in an in situ cell.

X-ray photoelectron spectra provides (K-Alpha, ThermoFisher, Waltham, MA, USA) a detailed insight into the composition of the Fe(III)-Alg and Mn(II)-Alg, as well the oxidation states of the ions. However, one should keep in mind that XPS is predominantly a surface analysis technique. The results for Fe can be seen in Figure 4. First of all, the atomic ratio between Fe and C (C:Fe) is fairly low compared to what we expected. C:Fe was measured at 68:1, while a pure Fe(III)-Alg should give a ratio between 18:1 and 12:1. A similar ratio was measured for C and Mn (63:1), which is much lower compared to the ratio of a pure Mn(II)-Alg (C:Mn 12:1). However, this could be due to the fact that XPS scans the surface of a sample and we expect ions to be wrapped inside the polysaccharide chains, inside the ‘egg-box structure’. The same explanation might apply to the relatively high sodium content. The sodium ion is not necessarily bound to an ‘egg-box structure’ and can therefore exist on the surface. It does show that the synthesis of the Fe(III)-Alg powder is not fully complete, meaning parts at the carboxyl groups are still compensated for by sodium ions, rather than being exchanged with iron ions. The Fe and Mn content in the alginates is approximately the same, which could imply a similar crosslinking efficiency. The chloride content in the Fe(III)-Alg is also surprising and it indicates to what extent Fe^3+^ is bound to the alginate chain. It would be sterically challenging for Fe^3+^ to bind to three carboxylate groups of the alginate. This result could indicate that Fe^3+^ binds to two carboxylates and one chloride anion. Iron is observed exclusively as Fe^3+^, while manganese shows more complex behaviour. Figure 5 shows the results of the Mn2p and Mn3s peaks before and after the charging of Mn(II)-Alg. Before charging, the peak separation ΔE = 6.14 eV and the satellite feature both confirm that the oxidation state of manganese is 2+. After charging, the oxidation state was again determined to be 2+. The difference between the two measurements is the lower amount of counts per second, which implies that when the oxidation state of the manganese increases, it leaves the electrode and dissolves most likely in the aqueous electrolyte.

#### 2.1.4. Mössbauer Spectroscopy

Mössbauer spectroscopy (Made in house at Delft University of Technology, Delft, Netherlands) measures the recoilless nuclear resonance fluorescence and can probe the chemical environment of an atom. This is based on the absorption and emission of gamma photons from deep orbitals that are sensitive to the ionic charge. Not all elements can be measured through Mössbauer spectroscopy; Fe can, but Mn cannot. A ‘free’ atom will have a lower quantum yield compared to a tightly packed atom, since some absorption energy of the ‘free’ atom will be changed to kinetic energy. In this paper, we use Mössbauer spectroscopy to determine the oxidation state of iron in our alginate samples. For Fe(III)-Alg, we could see 100% Fe^3+^ (Figure 5). For comparison, we also prepared a sample of Fe^2+^ ions in alginate giving Fe(II)-Alg, using Fe_2_SO_4 (aq)_ as the exchange solvent. It is seen that this Fe(II)-Alg oxidises partly to Fe(III)-Alg during the synthesis.

#### 2.1.5. Thermogravimetric Analysis (TGA)

**Figure 6:**
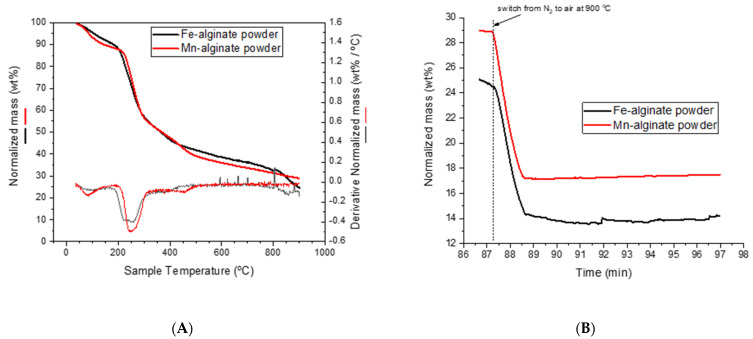
TGA of Fe(III)−Alg (**A**) and Mn(II)−Alg powder (**B**).

A thermogravimetric (TGA4000, PerkinElmer Waltham, MA, USA) analysis will give detailed information about the decomposition of the Fe(III)−Alg and Mn(II)−Alg at high temperature and various oxidative conditions. During the measurement, the weight of the compound is continuously measured (Figure 6). First, from room temperature to 175 °C, we see a decrease in sample mass of 10 wt% and 11 wt% for Fe(III)−Alg and Mn(II)−Alg, respectively, mainly caused by the release of water vapour, small organic molecules and other volatiles. Second, from 175 °C to 350 °C, decomposition of the alginate backbone and hydroxyl groups takes place. This further decreases the samples’ masses by 39 wt% and 37 wt%, respectively. Third, from 350 °C to 600 °C, decarboxylation takes place, reducing the sample masses further by 13 wt% and 15 wt%, respectively. Fourth, from 600 °C to 800 °C, some minor decomposition takes place. Finally, the samples are put under aerobic conditions, while maintaining the temperature at 900 °C where all remaining organics are combusted, leaving in the end 12 wt% residual mass from Fe(III)−Alg and 12 wt% of Mn(II)−Alg, reflecting iron oxide and manganese oxide, respectively.

Combining these results with the composition data obtained from XPS, we can approximate the theoretical capacities that these alginates can provide, i.e., 53 mAh/g for Fe(III)−Alg and 61 mAh/g for Mn(II)−Alg.

### 2.2. Electrochemical Experiments

#### 2.2.1. Electrode Preparation and Battery Assembly

To investigate the electrochemical performance of the transition metal alginates, two different electrodes were prepared: (i) with an alginate hydrogel and (ii) with the alginate powders.

i. The electrodes with the alginate hydrogels were made via dip coating of the hydrogel on carbon paper (CP), thus giving with an Fe(III)-Alg or Mn(II)-Alg hydrogel layer. The CP was first dipped into a 2% *w*/*w* Na-alginate solution and after 15 min of absorption, the CP with Na-alginate was then then placed in 0.5 M FeCl_3_ to form the Fe(III)-Alg hydrogel layer or in 0.5 M Mn(NO_3_)_2_ to form the Mn(II)-Alg hydrogel layer for another 15 min.
ii. The electrodes containing the alginate powders were made by casting a thin layer. This thin layer consisted of active material (synthesised Fe(III)-Alg powder or Mn(II)-Alg powder, 80 wt.%), a binder (PVDF, 10 wt.%) and conductive materials to provide a network for electrons to move freely (KS4 7 wt.%, carbon black (CB) 3 wt.%). NMP was used as the solvent. The ratio of the weight of all powders combined vs. the weight of NMP was approximately 1:1. This formed a thick slurry, which was then spread onto the current collector substrates with a doctor blade to achieve a 150 µm thick film. Aluminium and carbon paper were used as substrates. The substrates with the slurry coating were then dried in a vacuum oven and cut to the desired shape using a paper punch (diameter ~1.27 cm).

**Figure 7:**
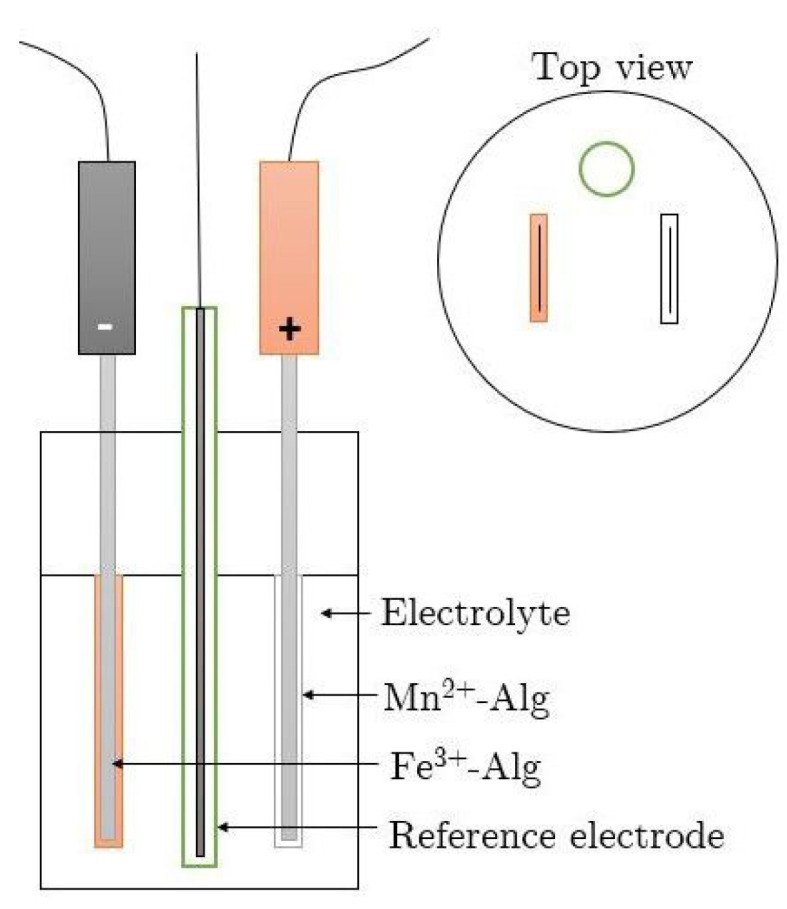
Battery Assembly for the Dip-Coated Electrode.

The two types of electrodes required different battery setups. The reference electrode used for both setups was Ag/AgCl and the electrolyte was Na-alginate with different concentrations running from 1–5 *w*/*w* Na-alginate, possibly with the co-addition of sodium chloride (0.25 M). The battery setup for the dip-coated electrodes was as shown in Figure 7.

**Figure 8:**
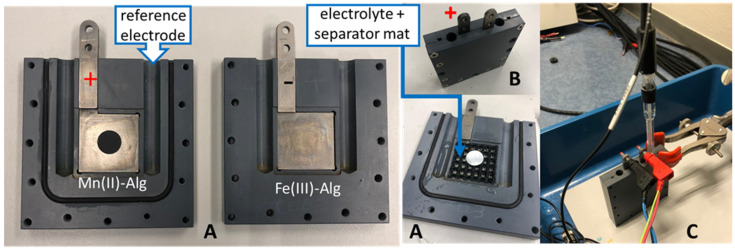
Test cell: (**A**) open test cell; (**B**) closed test cell with the electrodes; and (**C**) closed test cell with the reference electrode.

The thin-layer-coated electrode experiments were conducted in a test cell as shown in Figure 8. Here, two inert titanium plates acted as current collectors on which the electrodes were placed (Figure 8A). To prevent short circuiting between the electrodes, the electrolyte was placed in a mat between them (Figure 8A). The test cell was then closed (Figure 8B) and the reference electrode inserted (Figure 8C).

Both battery setups, as shown in Figure 7 and Figure 8, were then connected to a battery cycler (MACCOR 4000, Tulsa, OK, USA or Autolab Potentiostat PGSTAT302N device, Metrohm, Barendrecht, Netherlands) and measured via the standard software package to perform the electrochemical experiments.

#### 2.2.2. Cyclic Voltammetry (CV)

**Figure 9:**
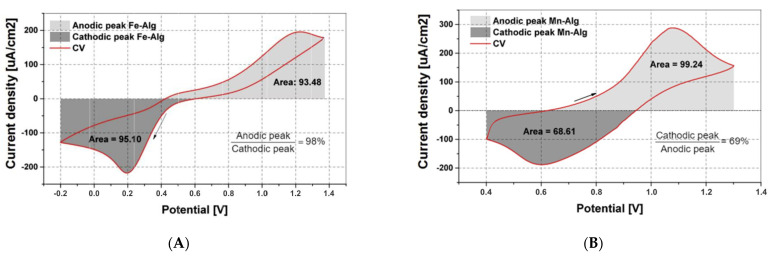
Cyclic voltammogram of (**A**) Fe(III)-Alg hydrogel. Scan rate n = 5 mV/s, 1% *w*/*w* Na-alginate electrolyte and (**B**) cyclic voltammogram of Mn(II)-Alg hydrogel. Scan rate n = 5 mV/s, 1% *w*/*w* Na-alginate electrolyte. The arrow shows the direction of the scan.

CV was used to investigate the reversibility of the hydrogels. The electrochemical cell with the dip-coated battery electrodes was connected to the Metrohm Autolab Potentiostat PGSTAT302N cycler (Autolab) for cycling. Both hydrogels, Mn(II)-Alg and Fe(III)-Alg, show reversibility (see Figure 9), implying that they can accept and donate electrons, though Fe(III)-Alg hydrogel exhibits a higher efficiency compared to Mn(II)-Alg hydrogel. The lower efficiency for Mn(II)-Alg hydrogel indicates that some of the electrons are lost because of unwanted side reactions. This supports the findings of the XPS that manganese after charging oxidises to higher states, which then leave the electrode. The Mn(II)-Alg hydrogel voltammogram shows the anodic peak (oxidation) at 1.07 V and the cathodic peak (reduction) at 0.6 V. The Fe(III)-Alg hydrogel voltammogram shows the cathodic peak (reduction) at 0.2 V and two anodic peak (oxidation) at 0.5 V and 1.2 V, respectively. This is indicative of the complex bonding nature of Fe^3+^, as was also observed from the XPS spectra.

#### 2.2.3. Charge/Discharge Profiling

**Figure 10:**
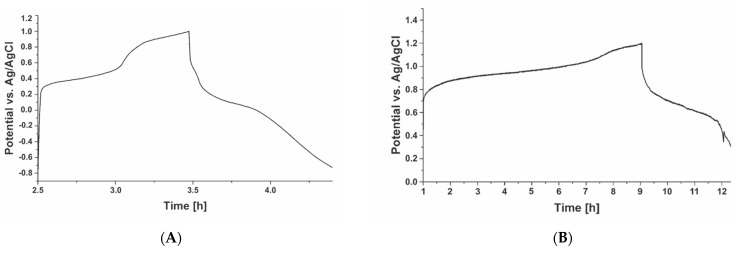
The cycle behaviour of (**A**) Fe(III)-Alg electrode from powder for 1 cycle at 0.1 mA in 1% *w*/*w* Na-alginate electrolyte and (**B**) Mn(II)-Alg electrode from powder for 1 cycle at 0.1 mA in 1% *w*/*w* Na-alginate electrolyte.

The electrochemical cell with the electrodes containing the alginate powders (Figure 8) was connected to the Maccor cycler to (dis)charge the cell at a constant current. Figure 10B shows that the Mn(II)-Alg powder has an oxidation potential of 0.98 V and reduction potential of 0.63 V. This is comparable with the potentials exhibited by the Mn(II)-Alg hydrogel (see Figure 9A); therefore, it is plausible that the same reaction occurs in both the hydrogel and powder, and an average reduction potential of the Mn^2+^/Mn^3+^ redox couple is approximately 0.8 V. The reduction potential for Fe(III)-Alg powder is seen at 2.0 V (Figure 10A) with two anodic peaks at 0.43 V (averaged from 2.5 h to 3 h) and 0.9 V (averaged from 3 h to 3.5 h). This is also comparable with potentials exhibited by the Fe(III)-Alg hydrogels (see Figure 9). The differences in the potential between the hydrogels and the powders can be attributed to differences in the internal resistances (impedance) of the systems; since the hydrogels are non-conductive, the resistance is greater, resulting in higher overpotentials. Hence, the actual redox potential is difficult to define because of the high and somewhat changing internal resistance of the electrode.

**Figure 11:**
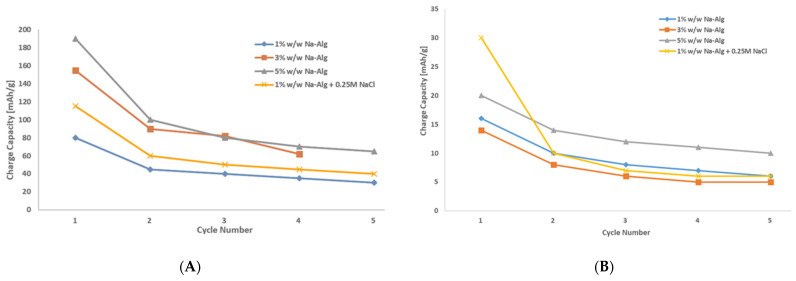
Specific capacity of: (**A**) Mn(II)-Alg electrodes from powder for different compositions of the electrolyte and (**B**) specific capacity Fe(III)-Alg electrodes from powder for different compositions of the electrolyte. I = 0.1 mA.

Figure 10 also gives the specific charge of Mn(II)-Alg powder and Fe(III)-Alg powder electrodes as 80 mAh/g and 16 mAh/g, respectively. For Mn(II)-Alg powder, this is 1.3 times higher than the TGA-derived capacity (61 mAh/g for one electron transfer), implying that the redox reaction may involve the transfer of two electrons (122 mAh/g for two electron transfer). Losses can be attributed to limitations by electrical and ionic conductivity. Figure 11A does show that the conductivity of Mn(II)-Alg powder is greatly influenced by ionic conductivity. The addition of more charge carriers (Na-Alg) in the electrolyte significantly improves the specific charge capacity of the Mn(II)-Alg electrode.

The specific capacity of Fe(III)-Alg powder (16 mAh/g) is much lower than the TGA capacity of 53 mAh/g. This suggests that the Fe(III)-Alg electrode is considerably affected by limited conductivity, which could be either ionic or electrical in nature. From the XPS analysis of Fe(III)-Alg, the amount of Cl^−^ ions is substantial; they are located closely and partially bound to the Fe^3+^ ion and, hence, form part of the overall internal resistance of the electrode. This suggests that the conductivity in the Fe(III)-Alg powder might be considerably affected by the Cl^−^ ionic conductivity. The literature indicates that trivalent cations form complex and irregular structures with alginates [7,8] as evidenced by the XPS spectra (see Figure 4): electrons “wrapped” up in this structure are less mobile, affecting the electronic conductivity. Figure 11B additionally illustrates that the addition of more charge carriers (Na-Alg) in the electrolyte does not significantly improve the specific charge capacity of Fe(III)-Alg powder (contrary to Mn(II)).

**Figure 12:**
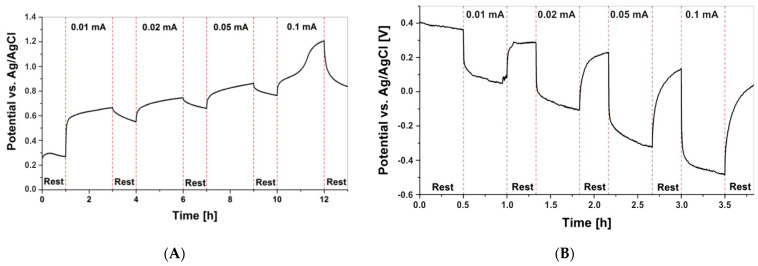
Stability of (**A**) Mn(II)-Alg powder for different values for I as indicated in the figure in 1% *w*/*w* Na-alginate electrolyte and (**B**) Fe(III)-Alg powder for different values for I as indicated in the figure in 1% *w*/*w* Na-alginate electrolyte.

To analyse the internal resistance (impedance), relaxation measurements were carried out, as presented in Figure 12. The Mn(II)-Alg cells were charged for 2 h and then allowed to rest for 1 h. Obviously, a significant drop in the voltage (about 0.1 V after 0.01 mA charge) is observed as a result of the charge overpotential, from which the impedance can be estimated to be 10 kΩ. A similar relaxation measurement was recorded for the Fe(III)-Alg cell, where the voltage drop is about three times higher, giving an impedance of about 30 kΩ. This significant difference in voltage drop has a clear impact on the obtained capacity, and thus it is obvious that the capacity of the Mn(II)-Alg found in our cell is much higher than the capacity found for the Fe(III)-Alg cell. Hence, the utilised capacity is strongly determined by the impedance of the cell. It is stressed, however, that we did not optimise these electrodes for the cells, and most likely we have to adjust the carbon-to-alginate-powder ratio, which will be explored further in the future.

**Figure 13:**
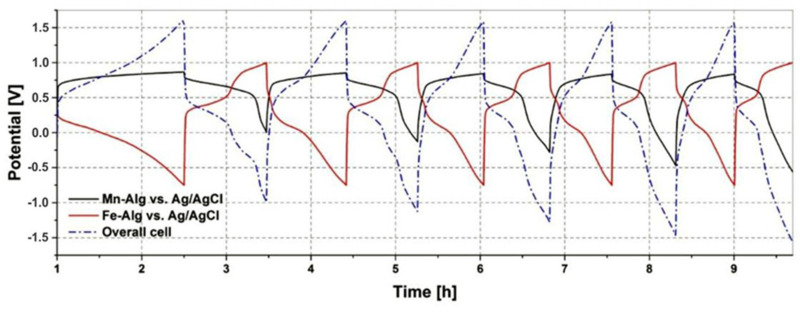
Cycle behaviour of a Mn(II)-Alg electrode from powder vs. an Fe(III)-Alg electrode from powder, 1% *w*/*w* Na-alginate electrolyte, at a charge/discharge current I = 0.1 mA.

Figure 13 shows the cycling of a Mn(II)-Alg powder electrode vs. an Fe(III)-Alg powder electrode, the arrangement of the intended battery of this research. Since the possible utilised capacity is much lower for the Fe(III)-Alg powder compared to the Mn(II)-Alg powder, the cut-off potentials are adjusted to the Fe(III)-Alg powder electrode to prevent electrolyte degradation. Here too, we can observe the huge influence of the impedance of the Fe(III)-Alg electrode. It is stressed that the negative overall potential is thus a result of the way the electrode potentials have been measured versus the reference electrode. Nevertheless, we see that the Fe(III)-Alg powder electrode is reversible, as the cycle behaviour stays the same with increasing cycling number. For the Mn(II)-Alg powder electrode, very stable behaviour is observed, but the capacity decreases as the cycle number increases. This is in line with the findings from Figure 10, Figure 11 and Figure 12. The increase in the charging potential seen in Figure 12A, at 11 h, is most likely a result of the Mn^3+^/Mn^4+^ redox couple. This is in contrast to the potential increase in the Fe(III)-Alg electrode in Figure 13 where the increase was a result of a change in internal resistance caused by Cl^−^ conductivity.

## 3. Conclusions

Fe(III) and Mn(II) alginate have been successfully synthetised and characterised; albeit, the ion exchange is not yet fully optimised. It appears that Fe(III)-Alg is charge compensated with one chloride ion and two alginate carboxylates, analogous to Ca-Alg and other divalent alginates. Similarly, Mn^2+^ behaves analogous to Ca^2+^ in alginate. Despite the room for further improvement, we have shown that both transition metal alginates can be used as electrode materials in a rechargeable battery with Fe(III)-Alg as the negative electrode and Mn(II)-Alg as the positive electrode, which is highly encouraging considering the low cost and low toxicity of this type of system. We have found a redox potential for Mn^2+^/Mn^3+^ in the Mn(II)-Alg electrode of 0.8 V versus Ag/AgCl. For the Fe(III)-Alg electrode, the internal resistances lead to a hard-to-interpret potential curve, which makes it difficult to define a proper reduction potential for Fe^2+^/Fe^3+^ in the Fe(III)-Alg electrode. In conclusion, although there is plenty of room for improvement, based on our current results, we find that Fe/Mn-alginate is a highly interesting platform for nontoxic, biocompatible and cheap electrochemical storage systems. Although not the topic of the current paper, we could remark that we might be able to use these two transition metal alginates as positive electrodes in a metal-ion battery, e.g., based on Mg or Zn.

## Data Availability

The data presented in this study are available on request from the corresponding author.
